# Wood's Household Magazine

**Published:** 1873-12

**Authors:** 


*Wood's Household Magazine.* Published by S. E. Shutes, New York city and Newburg, N. Y. at one dollar per year.

This is an entertaining and attractive journal. The December number, 1873, is replete with good reading, sketches, stories, poems &c, the contents embracing the following articles :

" A Better Country," Mary Hartwell ; An Engineer's Yarn, Albert Williams, Jr.; Our Party at Sea, Rev. J. S. Breckinridge ; Two Enthusiasts, H. M. Lewtral ; Presence of Mind, Rev. F. W. Holland ; Our Babies, D. A. Gorton, M. D. ; Blessedness of Riches, Tenoroon ; Hans Doodledee, Rudolph Mentel ; installment of Max Kromer, author of Jessica's First Prayer ; Codfish and Potatoes, Chapter II, by Eleanor Kirk ; Misery Jippeau, Chapter VII, VIII, by H. V. Osborne. In addition to these articles are several pretty poems, a charming little Cottage Design, and editorial departments embracing Our Housekeeper, Correspondence, Literary Notices, Laughing Stock, &c., &c. The engraving for this month is entitled "Old Folks."

For $1 50 a beautiful chromo is presented with the magazine for year 1874.

